# Polycythemia with elevated erythropoietin production in a patient with a urinary stone and unilateral hydronephrosis: a case report

**DOI:** 10.1186/s13256-023-03823-2

**Published:** 2023-03-09

**Authors:** Yuriko Hajika, Yuji Kawaguchi, Kenji Hamazaki, Yasuro Kumeda

**Affiliations:** Department of Internal Medicine, Minami Osaka Hospital, 1-18-18 Higashikagaya, Suminoe-Ku, Osaka, 559-0012 Japan

**Keywords:** Polycythemia, Secondary erythrocytosis, Hemoglobin, Erythropoietin, Hydronephrosis, Urinary stone

## Abstract

**Background:**

Absolute polycythemia can be primary or secondary. Erythropoietin-producing diseases (for example, hypoxia) are the major cause of secondary polycythemia. There are reports of polycythemia secondary to hydronephrosis. However, to our knowledge, there is no report on polycythemia secondary to hydronephrosis due to a urinary stone. Herein, we present a case of polycythemia with an elevated erythropoietin level in a patient with a urinary stone and unilateral hydronephrosis.

**Case presentation:**

A 57-year-old Japanese man presented with polycythemia and an elevated erythropoietin level. Erythropoietin accumulation was not due to erythropoietin secretion by a tumor as no obvious lesions were detected on contrast-enhanced computed tomography. Abdominal ultrasonography revealed a stone in the left urinary tract and renal hydronephrosis, and 2 weeks later, the patient underwent transurethral ureterolithotripsy without complications. Blood tests 2 weeks after transurethral ureterolithotripsy showed that the erythropoietin level had declined. Hemoglobin concentration decreased from 20.8 mg/dL before and immediately after transurethral ureterolithotripsy to 15.8 mg/dL 3 months after transurethral ureterolithotripsy. This case was diagnosed as erythropoietin elevation due to unilateral hydronephrosis with a urinary stone, resulting in polycythemia.

**Conclusions:**

Hydronephrosis is a common disease but is not often associated with polycythemia. Further studies are required to elucidate the mechanism and implications of elevated erythropoietin production in hydronephrosis.

## Background

Polycythemia is a blood disorder in which the hemoglobin (Hb) concentration or hematocrit is higher than normal. Its symptoms can be vague (for example, fatigue, headache, flushed complexion, and dizziness) or severe (for example, the formation of blood clots that can potentially cause heart attack, pulmonary embolism, or stroke) [1]. Other possible complications include enlargement of organs such as the liver or spleen [1, 2].

Polycythemia vera (primary polycythemia) is diagnosed when all three major criteria or the first two major criteria and the minor criterion are met. The major criteria include the following: (1) Hb concentration > 16.5 g/dL (men) or > 16.0 g/dL (women) and hematocrit > 49% (men) or > 48% (women); (2) bone marrow biopsy showing hypercellularity for age with trilineage growth; and (3) presence of *JAK2* or *JAK2* exon 12 mutation. The minor criterion is subnormal serum erythropoietin (EPO) level [3, 4]. The reported prevalence of polycythemia vera is approximately 22 cases per 100,000 people [5]. Secondary polycythemia is diagnosed in accordance with the World Health Organization definition of polycythemia vera using Hb reference values of 14.0–18.0 g/dL (men) and 12.0–16.0 g/dL (women). Chronic obstructive pulmonary disease, congenital heart disease, and severe pulmonary hypertension are the main causes of secondary polycythemia; however, data on the epidemiology of secondary polycythemia are lacking [2].

Polycythemia is classified as relative or absolute. Relative polycythemia is caused by reduced plasma volume, whereas absolute polycythemia usually results from EPO-producing diseases such as hypoxia or tumors [2]. Although the EPO level is elevated in secondary polycythemia, it is mostly decreased in polycythemia vera due to negative feedback [2, 3]. EPO is a hypoxia-inducible hematopoietic protein produced by fibroblast-like cells in the kidneys. It stimulates the proliferation of erythroid progenitor cells and promotes their gradual differentiation into erythrocytes. EPO production usually increases when oxygen pressure decreases in the kidney [6]. Hypoxia and EPO secretion by tumors can also lead to excessive EPO accumulation.

Hydronephrosis is the build-up of urine in one or both kidneys and their consequent swelling [7]. It can cause permanent kidney damage and kidney failure [8, 9] and often results from blockages in the urinary tract by kidney stones. Although there are reports of polycythemia secondary to hydronephrosis [10–12], to our knowledge, none has focused on polycythemia secondary to hydronephrosis due to a urinary stone. Herein, we present the first report of polycythemia with an elevated EPO level in a patient with a urinary stone and unilateral hydronephrosis. Unlike previous reports [10–12], we measured EPO levels before and after hydronephrosis treatment and linked the EPO production to polycythemia.

## Case presentation

**Fig. 1:**
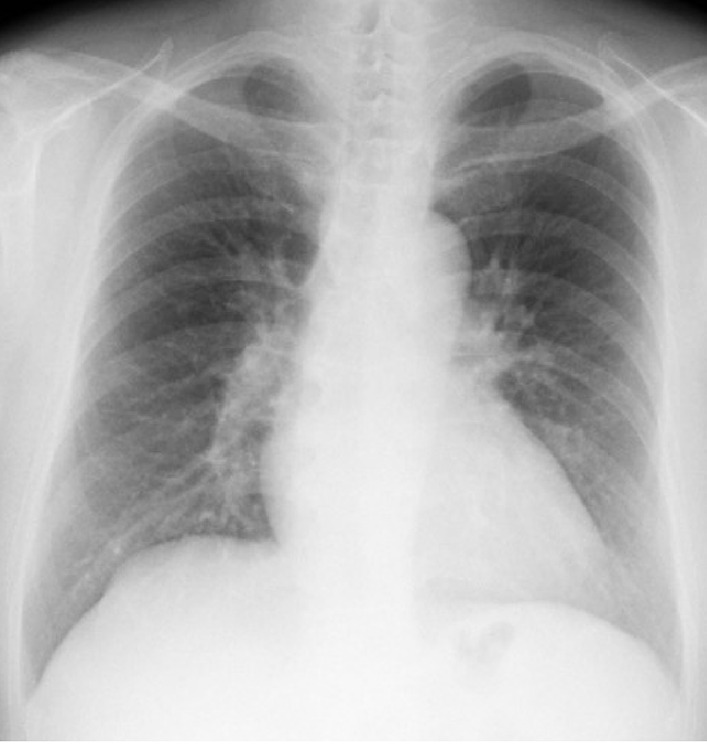
Chest radiograph on admission. The radiograph showed no significant findings

A 57-year-old Japanese man presented to our hospital with a red face, which was continually pointed out to him by his family in the preceding month. He had no notable past medical history, no family history of polycythemia, and had not been taking any medication. He had never smoked and did not take alcohol. He had a clerical job. His environmental history revealed no pertinent information regarding polycythemia. On presentation, his body mass index was 24.1 kg/m^2^, and his vital signs were as follows: body temperature 36.2 °C, pulse 74 beats per minute, blood pressure 132/74 mmHg, and percutaneous oxygen saturation level 99% (room air). Our hospital is located on the flatland in Osaka, Japan. On physical examination, there were no signs of hypoxia, such as shortness of breath, coughing, wheezing, or cyanosis. His breath sounds were clear bilaterally. Neurologic examination identified no abnormalities. A blood test indicated polycythemia; his Hb level was 20.8 g/dL (Table 1). No abnormality was observed in his chest radiograph (Fig. 1).

**Table 1:** Results of the laboratory examination at the first visit

Variable	Amount
Urine protein	(–)
Urine occult blood reaction	(–)
Urine glucose	(–)
Red blood cells in urine	1–4/HF
White blood cells in urine	1–4/HF
White blood cells	6100/μL
Red blood cells	622 × 10^4^/μL
Hemoglobin	20.8 g/dL
Hematocrit	59.5%
Platelets	21 × 10^4^/μL
Erythropoietin	70.5 mIU/mL
Total protein	7.8 g/dL
Albumin	4.5 g/dL
Blood urea nitrogen	11.2 mg/dL
Creatinine	0.74 mg/dL
Estimated glomerular filtration rate	84.5 mL/minute/1.73 m^2^
Lactate dehydrogenase	193 IU/L
Total bilirubin	1.1 mg/dL
Aspartate aminotransferase	28 IU/L
Alanine aminotransferase	32 IU/L
Amylase	53 IU/L
Creatine kinase	53 IU/L
Sodium	142 mEq/L
Potassium	3.8 mEq/L
Chlorine	103 mEq/L
Glucose	98 mg/dL
Glycated hemoglobin	5.7%
C-reactive protein	0.28 mg/dL

The reference value of hemoglobin in men is 14.0–18.0 g/dL

The reference value of erythropoietin is 4.2–23.7 mIU/mL

**Fig. 2:**
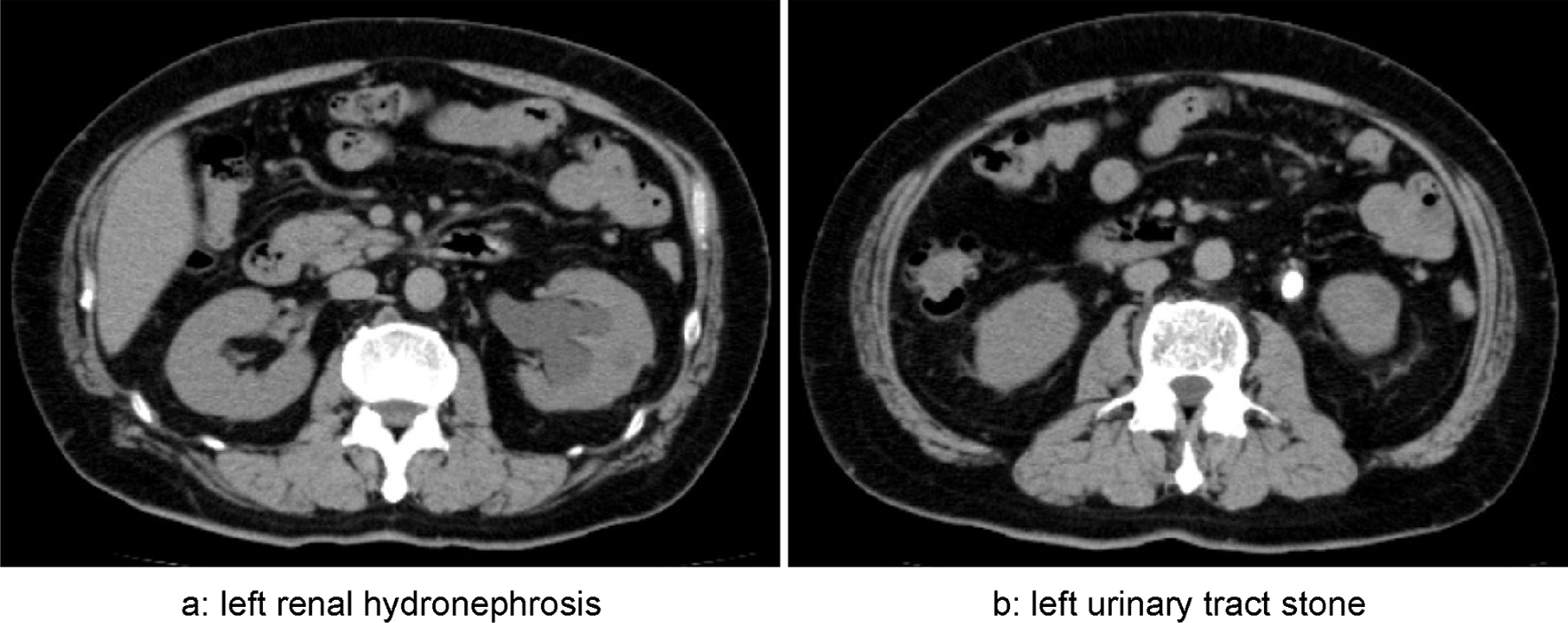
Plain thoracic, abdominal, and pelvic computed tomography. **a** left renal hydronephrosis. **b** left urinary tract stone

Because his EPO level was elevated (70.5 mIU/mL), polycythemia vera was unlikely. An EPO-producing tumor was considered; however, contrast-enhanced computed tomography revealed no obvious lesion. On further examination, including abdominal ultrasonography, a 17-mm stone in the left urinary tract and left renal hydronephrosis were evident (Fig. 2).

**Fig. 3:**
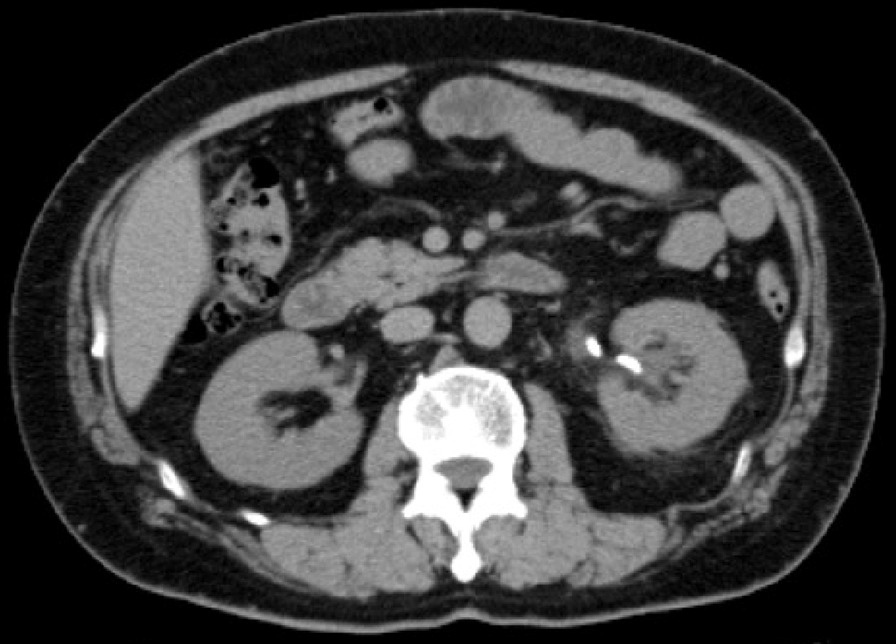
Plain thoracic, abdominal, and pelvic computed tomography. The white object in the left urinary tract is a double-J catheter, which was temporarily placed during transurethral ureterolithotripsy

Two weeks later, he was admitted to the urology department of our hospital for transurethral ureterolithotripsy (TUL) on a priority basis. The operation was completed without complications. During his 4 days of admission, he was prescribed 1.5 L of extracellular fluid and 2 g of cefotiam hydrochloride on the day of the procedure and the next day. He was prescribed 90 mg of carbazochrome sodium sulfonate hydrate for 7 days from the day of the procedure, and 2 weeks after the procedure, his EPO level had decreased from 70.5 mIU/mL to 7.5 mIU/mL. A follow-up examination showed reduced hydronephrosis (Fig. 3). In accordance with the 90-day half-life of red blood cells, his Hb level decreased from 20.8 mg/dL before and immediately after surgery to 15.8 mg/dL 3 months after surgery. Simultaneously, the redness in the patient’s face lessened. His Hb level was within the normal range (16.1 mg/dL) and his EPO remained low (5.4 mIU/mL) 6 months after surgery (Table 2).

**Table 2:** Changes in hemoglobin, hematocrit, and erythropoietin values over time

Variable	October (first visit)	November (before TUL)	November (after TUL)	February	April
Hemoglobin (g/dL)	20.8	20.2	20	15.8	16.1
Hematocrit (%)	59.5	59	56.8	46.3	46.8
Erythropoietin (mIU/mL)	70.5		7.5	9.6	5.4

*TUL* transurethral ureterolithotripsy

## Discussion and conclusions

We presented a case in which unilateral hydronephrosis with a urinary stone caused excessive EPO production, leading to polycythemia. Hydronephrosis involves the obstruction and dilation of the proximal urine passage by congenital abnormalities or masses such as tumors or stones. It is a common disease but is not often associated with polycythemia.

EPO is a hypoxia-inducible hematopoietic protein that stimulates the proliferation of erythroid progenitor cells and promotes their gradual differentiation into normoblasts and erythrocytes. EPO-producing cells are fibroblast-like cells located in the stroma around the proximal tubule at the boundary between the cortex and medulla. Hypoxia-inducible factor promotes the production of EPO by sensing a decrease in oxygen pressure, which is determined by the balance between oxygen supply from arteries and oxygen consumption in tissues [6].

Hypoxia increases EPO production, and some tumors secrete EPO; such tumors include renal cell carcinomas, hepatocellular carcinomas, hemangioblastomas, pheochromocytomas, and uterine fibroids [2]. The elevated level of EPO in the present case was not due to an EPO-producing tumor as no obvious lesions were found on contrast-enhanced computed tomography. Hypoxia was also excluded; although smokers have been reported to develop polycythemia, our patient had no smoking history.

The relevance of elevated EPO production in hydronephrosis remains unclear. In mice with unilateral hydronephrosis produced by obstructing a ureter, EPO maintained the peristaltic movement of the ureter [13]. Therefore, EPO should have maintained our patient’s ureteral function. Unlike what was observed in this case report, Eterović *et al*. [14] reported decreased serum EPO levels in 122 patients with partial unilateral urinary obstruction; stone sizes ranged from 15 mm to 26 mm. Two possibilities were considered in their report. The first was the possibility of hyperoxia, which reduces EPO production. However, this hypothesis was rejected by the authors due to decreased renal blood flow in the obstructed kidney. The second was the abnormal functioning of obstructed EPO-producing kidney cells. Although the EPO change differed between our report and that of Eterović *et al*., abnormal functioning of EPO-producing cells likely occurred in our case as well. Considering that EPO is produced in hypoxic situations, hypoxia around the EPO-producing cells might have been severe in our case. If excessive production of EPO had been caused by severe hypoxia in the cortical medulla border, it might have been an indication of early obstruction release. Hydronephrosis might have caused the difference between our case and that of Eterović *et al*.; however, the existence of hydronephrosis in their study is unknown. If timely treatment of the urinary stone and hydronephrosis had not been performed, hypoxia and consequent kidney cell damage might have occurred. Fortunately, renal function did not decline in this case.

To investigate changes in Hb levels in similar cases, we reviewed 88 cases in which patients with hydronephrosis and urinary stones underwent TUL in the past 3 years at our hospital. None of these cases involved polycythemia as in the present case. Similar to our case, 12 of the 88 cases showed a decrease in Hb levels after TUL, without causes such as apparent bleeding or medications; however, iatrogenic hemodilution due to the routine use of intravenous infusion after the procedure should be considered. It is unclear whether these cases fully resembled the present case in terms of Hb changes because there were no follow-up data regarding Hb levels. Measuring EPO and Hb levels before and at various times after TUL would aid in identifying the mechanisms underlying EPO production in hydronephrosis.

We present a case in which unilateral hydronephrosis with a urinary stone caused excessive EPO production and subsequent polycythemia. However, because hydronephrosis with polycythemia is uncommon, polycythemia due to hydronephrosis might not be considered. Further studies of suspected similar cases and measurement of EPO levels before and after hydronephrosis treatment are required to determine the mechanism, severity, and relevance of EPO production in hydronephrosis.

## Data Availability

Not applicable.

## Abbreviations

EPO: Erythropoietin
TUL: Transurethral ureterolithotripsy
Hb: Hemoglobin
